# Association of past 12-month sports injury history with hop limb symmetry index in physically active university students: a cross-sectional study of field-based functional asymmetry profiles

**DOI:** 10.3389/fpubh.2026.1868536

**Published:** 2026-07-03

**Authors:** Jie Gao, He Chen, Kaibin Fan

**Affiliations:** 1 Graduate School, Harbin Sport University, Harbin, China; 2 Ministry of Sports, Jiangsu Health Vocational College, Nanjing, Jiangsu, China; 3 School of Athletic Training, Nanjing Sport Institute, Nanjing, China

**Keywords:** field-based assessment, functional asymmetry, hop limb symmetry index, physically active university students, post-injury monitoring, sports injury history

## Abstract

**Background:**

Physically active university students are exposed to relatively high levels of sport and training, and unrecognized functional asymmetry may persist after sports injury. The hop limb symmetry index (hop LSI) is a feasible field-based assessment measure in university physical education and training settings and may help describe residual functional asymmetry at the group level and inform decisions about further functional monitoring.

**Objective:**

To examine whether past 12-month sports injury history is associated with lower hop LSI in physically active university students and to describe their lower-limb and trunk functional asymmetry profiles.

**Methods:**

This cross-sectional study included 263 physically active university students, including 202 participants without injury and 61 with injury. The primary exposure was past 12-month sports injury history, and the primary outcome was hop LSI. Hop LSI, the proportion of low LSI, ankle dorsiflexion asymmetry, Y-Balance Test (YBT) mean reach asymmetry, and side-bridge asymmetry were compared between groups. The primary analysis used ordinary least squares (OLS) linear regression with sequential adjustment for demographic characteristics, training exposure, activity background, low back pain, and functional asymmetry measures. Standard errors were estimated using cluster-robust standard errors based on class_id.

**Results:**

The injury group had lower hop LSI than the no-injury group (91.96 ± 3.63% vs. 94.88 ± 1.97%; mean difference = −2.92 percentage points, 95% confidence interval (CI): −3.89 to −1.96, *p* < 0.001). In supplementary threshold-based analyses, the injury group showed higher proportions of both moderate asymmetry (LSI < 92%, exploratory threshold) and marked asymmetry (LSI < 90%, conventional threshold) were 42.6% and 23.0% in the injury group and 7.9 and 2.0% in the no-injury group, respectively [odds ratio (OR) = 8.64 and 14.54]. The injury group also showed greater ankle dorsiflexion asymmetry (15.05% vs. 7.41%), YBT mean reach asymmetry (3.48 cm vs. 2.20 cm), and side-bridge asymmetry (12.82% vs. 9.80%). Regression analyses showed that past sports injury history was associated with lower hop LSI in the unadjusted model (*β* = −2.92, 95% CI: −3.74 to −2.11), the covariate-adjusted model (*β* = −3.13, 95% CI: −3.86 to −2.40), and the fully adjusted model further including functional asymmetry measures (*β* = −2.48, 95% CI: −3.29 to −1.66) (all *p* < 0.001). Findings remained consistent after excluding outliers and after additional adjustment for core_composite_z.

**Conclusion:**

Past 12-month sports injury history was associated with lower hop LSI and greater functional asymmetry in physically active university students. Combining hop LSI with multidimensional functional asymmetry assessment may help characterize current residual functional asymmetry profiles and guide post-injury functional monitoring in university physical education and training settings. These findings are descriptive and should not be interpreted as evidence of individual injury prediction; longitudinal studies are needed to evaluate predictive validity.

## 1 Introduction

Sports-related injury remains a persistent functional concern among physically active young adults, particularly among sports-major students and university students who participate in regular training. Previous studies in university populations have shown that physical activity-related injuries are not uncommon over a 12-month period, with lower-limb injuries accounting for a substantial proportion (1, 2). Collegiate sports surveillance data similarly indicate that the lower limb is one of the primary sites of both overall and severe sports injuries (3, 4). Past injury is also an important marker of subsequent lower-limb injury, and residual deficits in strength, neuromuscular control, dynamic balance, joint mobility, and bilateral performance symmetry may persist after return to regular exercise or training (5, 6). Beyond the injury event itself, residual deficits in strength, neuromuscular control, dynamic balance, joint mobility, and bilateral performance symmetry may persist even after return to regular exercise or training. The hop limb symmetry index (hop LSI) is commonly used as a field-based measure of bilateral lower-limb functional performance; however, previous studies suggest that symmetry-based measures alone may overestimate functional recovery or obscure persistent deficits in movement control and biomechanics (7–9). Therefore, for physically active university students who occupy an intermediate position between general university students and competitive athletes, characterizing residual functional asymmetry in relation to past injury has clear research relevance.

Common strategies to reduce lower-limb sports residual functional asymmetry include neuromuscular training, strength and balance training, mobility interventions, and structured return-to-sport assessment. Systematic reviews have shown that exercise-based injury prevention programmes can reduce sports residual functional asymmetry, particularly when strength and neuromuscular control components are included (10, 11). Return-to-sport consensus statements also emphasize that recovery assessment should not rely solely on time since injury but should incorporate multidimensional functional performance (12, 13). At the same time, field-based functional assessment is feasible in schools, sports teams, and physical education settings, but current evidence does not support using these measures for individual injury prediction (37, 38). Therefore, in the present cross-sectional context, these measures were used to characterize functional performance profiles and residual asymmetry associated with past injury, rather than to develop or validate a prediction model.

Functional asymmetry may be clinically relevant because it can reflect compensatory movement strategies during high-demand single-leg tasks (9). Landing biomechanics studies suggest that ankle position and ankle range of motion influence impact attenuation and joint loading, while altered hip-knee neuromuscular control and postural stability deficits during landing are associated with subsequent lower-limb residual functional asymmetry (14). Moreover, hop performance symmetry alone may mask persistent biomechanical asymmetry (9). Therefore, combining hop LSI with ankle dorsiflexion, YBT reach, and side-bridge asymmetry may better characterize injury-history–related movement strategy differences than using hop LSI as an isolated assessment measure.

Previous studies have examined the Y-Balance Test (YBT), single-leg hop tests, ankle dorsiflexion range of motion, trunk control, and past injury as separate factors. The YBT and Star Excursion Balance Test are commonly used to assess dynamic postural control and have been applied to characterize lower-limb functional asymmetry or functional deficits in athletic populations (39). Ankle dorsiflexion range of motion is closely related to dynamic balance and lower-limb motor function, and restricted dorsiflexion has also been examined in relation to lower-limb functional status in young athletic populations (15, 16). Trunk and core control may influence lower-limb functional performance, and prospective studies have linked core stability or trunk neuromuscular control with lower-limb residual functional asymmetry (17, 18). However, existing evidence is concentrated largely in competitive athletes, anterior cruciate ligament reconstruction populations, or single-sport samples. Many studies focus on individual functional tests, whereas fewer integrate hop LSI, ankle dorsiflexion asymmetry, YBT reach asymmetry, and trunk lateral endurance asymmetry within the same analytical framework. Among physically active university students, a population with both regular training exposure and non-elite sport characteristics, whether past 12-month injury history corresponds to residual functional asymmetry identifiable through field-based testing remains insufficiently examined (19).

Therefore, this cross-sectional study aimed to examine whether past 12-month sports injury history was associated with hop LSI and to characterize the accompanying lower-limb and trunk functional asymmetry profile in physically active university students. We hypothesized that students with a past 12-month injury history would show lower hop LSI and greater ankle dorsiflexion asymmetry, YBT reach asymmetry, and side-bridge asymmetry. Rather than establishing a causal pathway, demonstrating predictive validity, or developing an individual injury prediction model, this study sought to provide a field-based, multidimensional description of current functional asymmetry in relation to past injury history in a population frequently exposed to sport and training but less often evaluated through structured post-injury functional assessment.

## 2 Methods

### 2.1 Study design and reporting framework

This cross-sectional observational study included university students who regularly participated in physical activity or sport training. The study was designed to examine the association between past 12-month sports injury history and hop limb symmetry, and to further describe its relationship with lower-limb functional asymmetry. The manuscript was prepared in accordance with the Strengthening the Reporting of Observational Studies in Epidemiology (STROBE) checklist for cross-sectional studies (20). The study design, variable definitions, statistical analyses, and reporting of findings were structured to ensure transparency, reproducibility, and methodological consistency for a cross-sectional observational study.

### 2.2 Setting and participants

Participants were sports-major students and non-sports-major university students who had maintained regular exercise habits. They completed the questionnaire, anthropometric assessment, and lower-limb functional testing in batches according to class or teaching unit; therefore, class_id was treated as a potential clustering structure in the statistical analyses.

Eligibility criteria were as follows: (1) current university student status; (2) a background of regular physical activity or sport training; (3) ability to safely complete single-leg hop, ankle dorsiflexion range-of-motion, Y-Balance Test (YBT), side-bridge, and related functional performance tests; and (4) complete data for the primary exposure, primary outcome, and prespecified covariates. Regular exercise background included sports-major training, curriculum-based physical training, or long-term self-directed regular exercise participation.

Exclusion criteria were acute sports injury or pain at the time of testing that precluded safe completion of hop testing; recent lower-limb surgery or severe musculoskeletal disease; neurological or vestibular disorders that could affect balance, jumping, or neuromuscular control; missing data for the primary exposure, primary outcome, or key covariates; and failure to meet testing quality-control requirements or the presence of clearly abnormal records that could not be verified.

### 2.3 Variables and operational definitions

#### 2.3.1 Primary exposure

The primary exposure was past 12-month sports injury history (injury_12m), which indicated whether a participant had sustained any sport-related injury during the 12 months before testing. To ensure analytical consistency, all original codes were harmonized into two categories before analysis: no injury and injury, with no injury used as the reference group. This variable was therefore treated as a broad binary injury-history indicator. The available dataset did not contain complete and standardized information on anatomical injury location, injury type or diagnosis, severity, exact time since injury, rehabilitation status, or return-to-sport stage for all injured participants. Accordingly, the primary analyses were not stratified by these injury-specific characteristics, and all model estimates should be interpreted as average associations for a heterogeneous injury-history group.

#### 2.3.2 Primary outcome

The primary continuous outcome was the mean hop limb symmetry index (hop LSI; hop_lsi_mean_pct), expressed as a percentage, which reflected bilateral symmetry across four single-leg hop tasks: the single hop for distance, triple hop for distance, crossover hop for distance, and 6-m timed hop. For each hop task, side-specific performance was first calculated as the mean of two valid formal trials for each limb. For the three distance-based hop tasks, task-specific LSI was calculated as the lower mean distance divided by the higher mean distance and multiplied by 100%. For the 6-m timed hop, because a shorter time indicates better performance, task-specific LSI was calculated as the shorter mean time divided by the longer mean time and multiplied by 100%. The four task-specific LSI values were then averaged to obtain the mean hop LSI. Lower mean hop LSI values indicated greater interlimb asymmetry.

#### 2.3.3 Secondary outcomes

Binary low-LSI outcomes were derived from the primary continuous outcome and included hop_lsi_lt92 and hop_lsi_lt90. The variable hop_lsi_lt92 was defined as hop_lsi_mean_pct < 92 and was used as the primary binary secondary outcome, whereas hop_lsi_lt90 was defined as hop_lsi_mean_pct < 90 and was used as a more stringent threshold in sensitivity analysis. Both binary outcomes were used to evaluate the association between past sports injury history and low hop symmetry. The LSI < 90% threshold was used as a conventional reference for marked hop asymmetry, consistent with commonly used limb symmetry criteria in hop-test and return-to-sport literature. The LSI < 92% threshold was used as an exploratory, more sensitive classification to describe moderate asymmetry in this physically active university sample. These thresholds were not intended as diagnostic or prospective injury-risk cutoffs; continuous hop LSI was retained as the primary outcome.

#### 2.3.4 Functional asymmetry measures

Functional asymmetry measures were used to describe interlimb differences related to lower-limb mobility, dynamic balance, and trunk lateral endurance. Prespecified asymmetry variables included ankle dorsiflexion asymmetry percentage (ankle_asym_pct), absolute ankle dorsiflexion asymmetry (ankle_asym_cm), YBT mean reach asymmetry (ybt_asym_mean_cm), YBT anterior, posteromedial, and posterolateral reach asymmetry (ybt_ant_asym_cm, ybt_pm_asym_cm, and ybt_pl_asym_cm), and side-bridge asymmetry (side_bridge_asym_pct) (41, 42, 43). The final multivariable model prespecified ankle_asym_pct, ybt_asym_mean_cm, and side_bridge_asym_pct as representative asymmetry measures for ankle mobility, dynamic balance, and core lateral endurance, respectively.

#### 2.3.5 Covariates and sensitivity variables

Covariates were selected *a priori* based on previous literature, the study design, and plausible confounding structure. They included sex, age_years, body mass index (BMI; bmi), weekly training duration (training_hours_week), training experience (training_years), activity group (activity_group), and low back pain in the past 3 months (low_back_pain_3m). The variable class_id was not treated as a conventional covariate but was used to represent class- or teaching-unit-level clustering and served as the clustering unit for robust standard error estimation. The variable sim_outlier_flag was used for the outlier-exclusion sensitivity analysis, whereas core_composite_z was used only in the additional core composite function-adjusted model to assess whether the association between injury history and hop LSI remained stable after accounting for overall core function.

### 2.4 Measurement procedures

#### 2.4.1 General testing procedure

All tests were conducted at a standardized testing site and followed a prespecified testing sequence. The testing order was not randomized; instead, all participants completed the assessments in the same order to improve procedural consistency across testing batches. Before testing, the research staff explained the procedures, task requirements, and safety considerations to the participants. Participants completed a standardized warm-up and familiarization trials before formal testing. All assessments were administered and recorded by trained evaluators to reduce inter-rater variability. Appropriate rest intervals were provided between tests to limit the influence of fatigue on functional performance. For tests involving bilateral comparisons, both limbs were assessed separately, and the order of side testing was kept consistent across participants. The dominant limb was defined as the limb that the participant preferred to use when kicking a ball; however, hop LSI was calculated using the lower-performing limb divided by the higher-performing limb, rather than using limb dominance. When an obvious performance error, failure to follow instructions, or abnormal data recording occurred, the evaluators reviewed the trial according to prespecified quality-control criteria and repeated the test when appropriate. All raw data were recorded on site and checked after testing for completeness and logical consistency.

#### 2.4.2 Injury history and participant characteristics

Participant characteristics and training background were collected using a standardized recording form, including age, sex, height, body mass, training experience, and weekly training duration; body mass index (BMI) was calculated from height and body mass. Activity group, sports-major status, and curriculum track were recorded according to participants’ academic and training backgrounds. Past 12-month sports injury history and low back pain in the past 3 months were obtained using a standardized questionnaire or recording procedure and were used to define the primary exposure and a prespecified covariate, respectively. Injured side (injured_side), when available, was summarized only as a descriptive and exploratory injury characteristic and was not included in the primary regression models. Because injured side does not capture anatomical injury location, injury type or diagnosis, severity, time since injury, rehabilitation status, or return-to-sport stage, it was not considered a substitute for detailed injury-specific stratification.

#### 2.4.3 Hop performance and hop LSI

Hop performance was assessed bilaterally using four single-leg hop tasks: the single hop for distance, triple hop for distance, crossover hop for distance, and 6-m timed hop. All hop tests were performed after standardized instructions, demonstration, warm-up, and familiarization. For each hop task and each limb, participants completed two valid formal trials. Thus, for each participant, two formal trials were recorded for the right limb and two formal trials for the left limb in each of the four hop tasks.

For the single hop, triple hop, and crossover hop, hop distance was recorded in centimeters, and a longer distance indicated better performance. For the 6-m timed hop, completion time was recorded in seconds, and a shorter time indicated better performance. The side-specific score for each hop task was calculated as the mean of the two valid formal trials for that limb (40). A trial was considered invalid and repeated if the participant lost balance, failed to maintain the landing position, used the contralateral limb for support, touched down with the opposite limb, or did not follow the test instructions.

The hop limb symmetry index (hop LSI) was calculated from the side-specific mean values. For distance-based hop tasks, task-specific LSI was calculated as the lower-performing limb divided by the higher-performing limb and multiplied by 100%. For the 6-m timed hop, the direction of the metric was reversed by calculating the shorter mean time divided by the longer mean time and multiplying by 100%, so that lower LSI values consistently represented greater interlimb asymmetry. The mean hop LSI (hop_lsi_mean_pct), used as the primary continuous outcome, was calculated as the arithmetic mean of the four task-specific LSI values.

#### 2.4.4 Functional asymmetry measures

The Y-Balance Test Lower Quarter (YBT-LQ) was used to assess dynamic balance in the anterior, posteromedial, and posterolateral directions. The YBT-LQ was performed barefoot according to a standardized protocol. For each trial, participants stood on the tested limb at the center of the YBT testing grid, with the toes positioned behind the starting line, and reached with the contralateral limb as far as possible in the required direction while maintaining balance and returning to the starting position under control. The testing order was fixed for all participants as follows: right anterior, left anterior, right posteromedial, left posteromedial, right posterolateral, and left posterolateral.

Before formal testing, participants completed six familiarization trials in each direction for each limb. During formal testing, three valid trials were recorded for each direction on each limb. A trial was considered invalid and repeated if the participant failed to maintain single-leg stance, moved or repositioned the stance foot, touched the floor with the reaching foot before returning to the starting position, kicked or used the reach indicator for support, reported pain, or failed to return the reaching foot to the starting position under control.

The greatest valid reach distance from the three formal trials was retained for each direction and each limb. Limb length was measured from the anterior superior iliac spine to the distal end of the medial malleolus. Direction-specific normalized reach distance was calculated as: reach distance/limb length × 100%. The YBT-LQ composite score for each limb was calculated as: [(anterior reach distance + posteromedial reach distance + posterolateral reach distance)/(3 × limb length)] × 100%.

In the present study, YBT asymmetry was used as a functional asymmetry variable. Directional YBT asymmetry was calculated as the absolute side-to-side difference in reach distance for the anterior, posteromedial, and posterolateral directions, expressed in centimeters. The three directional asymmetry values were then averaged to obtain YBT mean reach asymmetry (ybt_asym_mean_cm). Therefore, YBT composite scores were normalized to limb length and expressed as percentages, whereas the YBT asymmetry variable used in the regression models represented absolute side-to-side reach differences in centimeters.

#### 2.4.5 Other functional measures

In addition to hop and asymmetry measures, other functional performance variables were recorded to describe the overall functional profile across injury-history groups. The Landing Error Scoring System (LESS) was used to assess landing movement quality, with higher scores indicating more landing errors or poorer movement control. Simple reaction time was recorded in milliseconds as an indicator of basic response speed.

The lower-extremity function z-score (lower_ext_function_z) and global function z-score (global_function_z) were calculated from prespecified functional performance measures after standardization. The direction of all component variables was harmonized before aggregation so that higher scores indicated better functional performance. These measures were used primarily for descriptive comparisons and supplementary analyses and were not treated as the primary outcome.

### 2.5 Bias control and data quality

To reduce measurement bias, all tests were conducted by the same assessment team using standardized procedures. Assessors received training before formal testing and used the same test instructions, demonstrations, and recording criteria. The testing site, sequence, warm-up procedure, and rest intervals were kept consistent to reduce the influence of environmental variation, learning effects, and fatigue accumulation on functional performance. Because participants were tested by class or teaching unit, observations within the same unit may have been correlated. Therefore, class_id was used as the clustering unit for cluster-robust standard errors in regression analyses. When clustering information was unavailable or the number of clusters was insufficient, HC3 robust standard errors were used as an alternative to improve the robustness of standard error estimation.

Trial-level processing rules were applied before derived variables were calculated. For hop tests, the two valid formal trials for each limb and each task were averaged to obtain side-specific hop performance. For the YBT-LQ, the greatest valid reach distance from three formal trials in each direction and each limb was retained before calculating normalized composite scores and side-to-side asymmetry variables.

### 2.6 Study size

This study was based on an existing cross-sectional dataset and included 263 physically active university students, comprising 202 participants without past 12-month sports injury history and 61 participants with past 12-month sports injury history. Because the primary outcome was continuous hop LSI, the study size was considered appropriate for the prespecified multivariable linear regression models.

Because no formal *a priori* sample size calculation was conducted for this existing dataset, a *post hoc* precision/detectable-effect analysis was performed for the primary continuous outcome, hop LSI. This analysis used the observed group sizes and variability to estimate the minimum detectable absolute between-group difference in hop LSI. The purpose of this analysis was to contextualize the precision of the primary continuous outcome analysis rather than to redefine statistical significance. Binary low-LSI outcomes based on the LSI < 92% and LSI < 90% thresholds were treated as secondary and sensitivity outcomes. Because the number of events was limited, especially for the LSI < 90% outcome, Firth penalized logistic regression was additionally used as a small-event sensitivity analysis for the binary low-LSI outcomes.

### 2.7 Statistical analysis

All statistical analyses were performed in R. Two-sided tests were used, with the significance level set at *α* = 0.05. Continuous variables were reported as mean ± standard deviation (SD), and categorical variables as *n* (%). After participants were classified into the no injury and injury groups according to past 12-month sports injury history, continuous variables were compared using Welch’s *t*-test, and categorical variables were compared using the *χ*^2^ test or Fisher’s exact test. Standardized mean difference (SMD) was also calculated to evaluate between-group differences. Functional performance and asymmetry measures were compared descriptively between groups; continuous outcomes were reported with mean differences and 95% confidence intervals (CIs), and binary low-LSI outcomes were reported with odds ratios (ORs) and 95% CIs.

The primary analysis used ordinary least squares (OLS) linear regression with hop_lsi_mean_pct as the outcome. Models were constructed sequentially as an unadjusted model, a model adjusted for demographic characteristics and training exposure, and a fully adjusted model additionally including ankle dorsiflexion asymmetry, YBT mean reach asymmetry, and side-bridge asymmetry. Regression results were reported as *β* coefficients, 95% CIs, *p* values, and model fit indices. To account for clustering by class or teaching unit, standard errors were preferentially estimated using cluster-robust standard errors based on class_id; when clustering conditions were insufficient, HC3 robust standard errors were used. Secondary and sensitivity analyses included logistic regression for binary low-LSI outcomes, exclusion of outlier-flagged observations, additional adjustment for core_composite_z, interaction testing, Spearman correlation analysis, and final model diagnostics. Holm correction was applied for multiple testing in correlation analyses.

Multicollinearity in the fully adjusted model was assessed using variance inflation factors (VIFs). For categorical variables, generalized VIFs were examined when applicable. VIF or adjusted GVIF values below 5 were considered to indicate no serious multicollinearity. Spearman correlations among hop LSI and selected functional asymmetry indicators were also calculated to assess potential redundancy among variables.

Because this study was based on an existing cross-sectional dataset, no formal *a priori* sample size calculation was conducted. To address study-size considerations, a *post hoc* precision/detectable-effect analysis was performed for the primary continuous outcome, hop LSI. This analysis used the observed no-injury and injury group sizes and the observed variability of hop LSI to estimate the minimum detectable absolute between-group difference. The analysis was used only to contextualize the study size and precision of the primary continuous outcome analysis.

For the secondary binary low-LSI outcomes, event numbers were limited, particularly for the LSI < 90% outcome. Therefore, Firth penalized logistic regression was conducted as a small-event sensitivity analysis for both LSI < 92% and LSI < 90%. The Firth models used past 12-month sports injury history as the primary exposure and were estimated under two specifications: a main covariate-adjusted model adjusted for sex, age, BMI, weekly training duration, training experience, activity group, and low back pain in the past 3 months, and an exploratory functional-adjusted model additionally including ankle dorsiflexion asymmetry, YBT mean reach asymmetry, and side-bridge asymmetry. Results from Firth penalized logistic regression were reported as odds ratios with 95% confidence intervals and *p* values. These analyses were interpreted as sensitivity analyses for the threshold-based secondary outcomes and were not considered primary evidence.

### 2.8 Ethics and consent

The study protocol was reviewed and approved by the Ethics Committee of Nanjing Sport Institute (approval number: RT2025-13). Before testing, all participants received information about the study purpose, testing procedures, potential risks, and data use, and provided written informed consent voluntarily. Study data were anonymized before analysis and used only for the statistical analyses related to this study. Participants were free to withdraw without any effect on their academic or training arrangements.

## 3 Results

### 3.1 Participant characteristics

A total of 263 physically active university students were included in the final analysis, including 202 participants without past 12-month sports injury history and 61 who reported past 12-month sports injury history. Among participants with past 12-month injury history, injured-side information was used only for descriptive and exploratory characterization. No subgroup analysis by anatomical injury location, injury type or diagnosis, severity, time since injury, rehabilitation status, or return-to-sport stage was performed because these variables were not consistently available. Therefore, subsequent comparisons represent average differences between participants with and without any past 12-month sports injury history. The overall sample had a mean age of 20.39 ± 1.01 years and a body mass index (BMI) of 22.53 ± 1.95 kg/m^2^. Age distribution was similar between groups, whereas the injury group showed more apparent differences in sex distribution, BMI, sport background, and training exposure. Compared with the no-injury group, the injury group had a higher proportion of male participants (62.3% vs. 46.5%) and a higher BMI (23.03 ± 1.90 vs. 22.38 ± 1.94 kg/m^2^). Regarding sport background, a larger proportion of participants in the injury group were sports-major students or classified as SportsMajor (75.4% vs. 42.6%), and the distribution of programme or curriculum track also differed between groups. Training exposure was greater in the injury group, with longer weekly training duration (10.70 ± 2.77 vs. 8.38 ± 3.20 h/week) and longer training experience (6.27 ± 1.98 vs. 5.31 ± 1.93 years), indicating that the two groups were not fully balanced in sport participation intensity and accumulated training exposure.

For musculoskeletal health status, the prevalence of low back pain in the past 3 months was higher in the injury group than in the no-injury group (16.4% vs. 7.9%), although the between-group difference approached but did not reach conventional statistical significance. The proportion of observations flagged as simulated outliers differed little between groups (6.6% vs. 4.5%). Overall, the injury group had not only a greater burden of past injury but also a more concentrated sports-major background and higher training exposure. These baseline differences provided the basis for subsequent multivariable adjustment. Participant characteristics are shown in Table 1.

**Table 1:** Participant characteristics according to injury history in the past 12 months.

Characteristic	Overall (*N* = 263)	No injury (*n* = 202)	Injury (*n* = 61)	*p* value	SMD
Sex				0.031	0.320
Female	131 (49.8)	108 (53.5)	23 (37.7)		
Male	132 (50.2)	94 (46.5)	38 (62.3)		
Age, years	20.39 ± 1.01	20.35 ± 0.99	20.50 ± 1.06	0.343	0.142
BMI, kg/m^2^	22.53 ± 1.95	22.38 ± 1.94	23.03 ± 1.90	0.022	0.338
Activity group				<0.001	0.708
Regular active	131 (49.8)	116 (57.4)	15 (24.6)		
Sports major	132 (50.2)	86 (42.6)	46 (75.4)		
Program track				<0.001	0.749
Recreation runner	33 (12.5)	30 (14.9)	3 (4.9)		
Intramural sports	33 (12.5)	30 (14.9)	3 (4.9)		
Fitness club	65 (24.7)	56 (27.7)	9 (14.8)		
Track field	26 (9.9)	17 (8.4)	9 (14.8)		
Soccer	25 (9.5)	14 (6.9)	11 (18.0)		
Basketball	26 (9.9)	16 (7.9)	10 (16.4)		
Physical education	55 (20.9)	39 (19.3)	16 (26.2)		
Training hours/week	8.92 ± 3.25	8.38 ± 3.20	10.70 ± 2.77	<0.001	0.777
Training years	5.53 ± 1.98	5.31 ± 1.93	6.27 ± 1.98	0.001	0.491
Low back pain in past 3 months				0.052	0.261
No	237 (90.1)	186 (92.1)	51 (83.6)		
Yes	26 (9.9)	16 (7.9)	10 (16.4)		
Simulation outlier flag				0.507	0.092
No	250 (95.1)	193 (95.5)	57 (93.4)		
Yes	13 (4.9)	9 (4.5)	4 (6.6)		

Values are mean ± SD for continuous variables and *n* (%) for categorical variables. Percentages are column percentages. Missing values, if present, are shown but excluded from hypothesis tests. Welch *t*-test was used for continuous variables. Chi-square test was used for categorical variables; Fisher exact test was used automatically when expected cell counts were insufficient. SMD, standardized mean difference.

### 3.2 Functional performance and asymmetry profiles according to injury history

**Figure 1:**
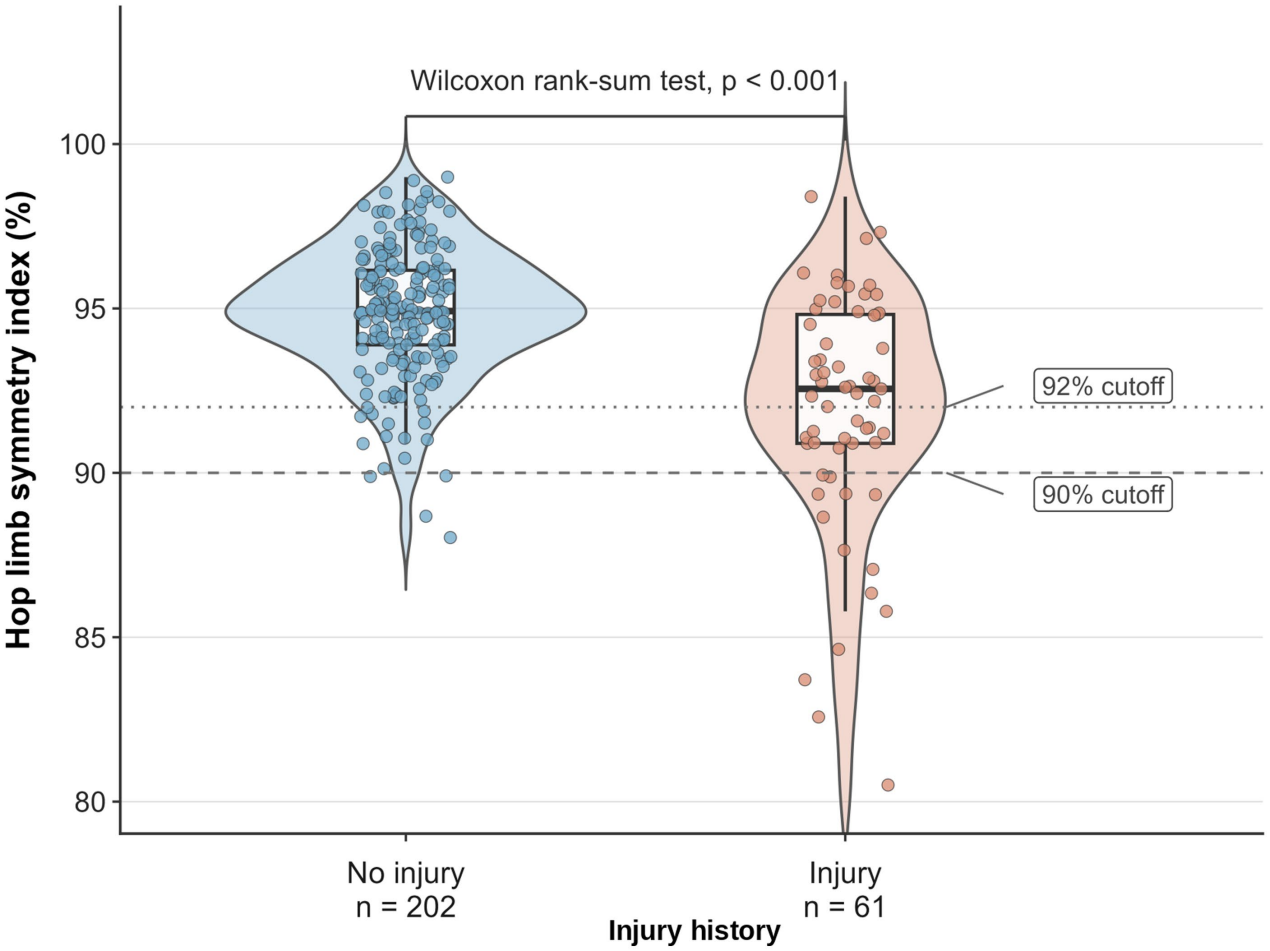
Distribution of hop limb symmetry index according to injury history.

For the primary outcome, participants with past 12-month sports injury history showed lower hop limb symmetry index (hop LSI). The no-injury group had a hop LSI of 94.88 ± 1.97%, whereas the injury group had a hop LSI of 91.96 ± 3.63%; the between-group mean difference was −2.92 percentage points (95% CI: −3.89 to −1.96, *p* < 0.001). This difference indicated that the injury group had lower overall hop limb symmetry than the no-injury group, in a direction consistent with the primary hypothesis (Table 2). When classified according to reference thresholds, the injury group had a markedly higher proportion of low LSI. Using the 92% threshold, 42.6% of participants in the injury group were below the threshold, compared with 7.9% in the no-injury group (OR = 8.64). Using the 90% threshold, 23.0% of participants in the injury group were below the threshold, compared with 2.0% in the no-injury group (OR = 14.54). Figure 1 shows that the hop LSI distribution in the injury group shifted toward lower values, with more participants falling below the 92 and 90% reference lines.

**Table 2:** Functional performance and asymmetry profiles according to injury history.

Characteristic	Overall (*N* = 263)	No injury (*n* = 202)	Injury (*n* = 61)	Mean difference/OR	95% CI	*p* value
Hop LSI, %	94.20 ± 2.74	94.88 ± 1.97	91.96 ± 3.63	−2.92	−3.89 to −1.96	<0.001
Hop LSI < 90%, *n* (%)	18 (6.8)	4 (2.0)	14 (23.0)	14.54	4.31 to 63.43	<0.001
Hop LSI < 92%, *n* (%)	42 (16.0)	16 (7.9)	26 (42.6)	8.64	4.20 to 17.74	<0.001
Ankle asymmetry, %	9.19 ± 7.87	7.41 ± 6.04	15.05 ± 10.13	7.64	4.91 to 10.36	<0.001
Ankle asymmetry, cm	1.01 ± 0.85	0.83 ± 0.67	1.61 ± 1.08	0.78	0.49 to 1.07	<0.001
YBT asymmetry, mean, cm	2.49 ± 1.43	2.20 ± 1.08	3.48 ± 1.93	1.28	0.77 to 1.80	<0.001
YBT anterior asymmetry, cm	2.61 ± 2.00	2.27 ± 1.80	3.74 ± 2.21	1.47	0.85 to 2.08	<0.001
YBT posteromedial asymmetry, cm	2.38 ± 2.02	2.14 ± 1.56	3.20 ± 2.95	1.07	0.28 to 1.85	0.009
YBT posterolateral asymmetry, cm	2.48 ± 2.01	2.18 ± 1.65	3.49 ± 2.67	1.31	0.59 to 2.03	<0.001
Side bridge asymmetry, %	10.50 ± 8.11	9.80 ± 7.36	12.82 ± 9.93	3.02	0.29 to 5.75	0.031
Single hop, cm	173.68 ± 15.98	175.19 ± 16.23	168.65 ± 14.13	−6.54	−10.78 to −2.30	0.003
Triple hop, cm	538.09 ± 55.19	542.32 ± 56.17	524.10 ± 49.74	−18.21	−33.06 to −3.36	0.017
Crossover hop, cm	493.61 ± 46.17	498.16 ± 46.45	478.57 ± 42.21	−19.59	−32.11 to −7.07	0.002
Timed 6-m hop, s	2.07 ± 0.13	2.07 ± 0.13	2.10 ± 0.12	0.03	−0.00 to 0.06	0.083
Lower-extremity function *z*-score	0.00 ± 0.76	0.08 ± 0.77	−0.25 ± 0.67	−0.33	−0.53 to −0.13	0.001
Global function *z*-score	0.00 ± 0.70	0.05 ± 0.72	−0.18 ± 0.60	−0.24	−0.42 to −0.05	0.012
LESS score	3.95 ± 1.87	3.90 ± 1.82	4.10 ± 2.04	0.20	−0.38 to 0.77	0.499
Simple reaction time, ms	283.33 ± 46.09	282.09 ± 47.65	287.43 ± 40.57	5.33	−6.91 to 17.58	0.390

Values are mean ± SD for continuous variables and *n* (%) for binary variables. For binary variables, *n* (%) indicates the number and percentage of participants meeting the specified threshold. Mean difference was calculated as Injury minus no injury. OR indicates the odds ratio for the binary event in the Injury group compared with the no injury group. Continuous variables were compared using Welch *t*-test. Binary variables were analyzed using logistic regression; Fisher exact test was used when sparse cell counts were present.

**Figure 2:**
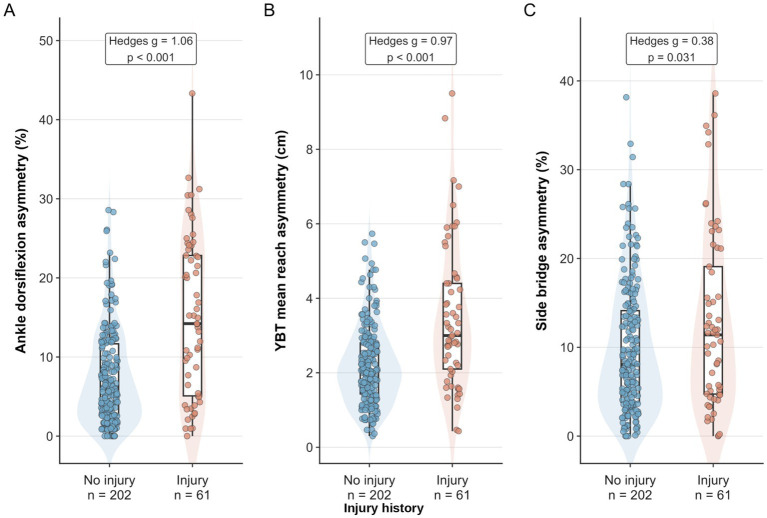
Functional asymmetry profiles according to injury history: **(A)** Ankle dorsiflexion asymmetry (%); **(B)** YBT mean reach asymmetry (cm); **(C)** Side bridge asymmetry (%).

The injury group also showed greater functional asymmetry. Ankle dorsiflexion asymmetry was higher in the injury group than in the no-injury group (15.05% vs. 7.41%). Y-Balance Test (YBT) mean reach asymmetry was 3.48 cm in the injury group and 2.20 cm in the no-injury group, and side-bridge asymmetry was 12.82 and 9.80%, respectively. Figure 2 further shows that the between-group differences were most evident for ankle dorsiflexion asymmetry and YBT mean reach asymmetry, with Hedges’ *g* values of 1.06 (*p* < 0.001) and 0.97 (*p* < 0.001), respectively. The difference in side-bridge asymmetry was smaller but still observable (Hedges’ *g* = 0.38, *p* = 0.031). These findings indicate that past injury history was associated not only with lower hop limb symmetry but also with higher levels of lower-limb functional asymmetry. Other functional performance measures showed a generally similar pattern. Compared with the no-injury group, the injury group had shorter single-hop, triple-hop, and crossover-hop distances, as well as lower lower-extremity and global function *z*-scores. In contrast, no clear between-group differences were observed for Landing Error Scoring System (LESS) score or simple reaction time. Overall, past 12-month sports injury history was associated with lower hop LSI, a higher proportion of low LSI, and more pronounced lower-limb functional asymmetry.

### 3.3 Multivariable association between injury history and hop LSI

Linear regression analyses showed a stable association between past 12-month sports injury history and lower hop LSI. In the unadjusted model, the injury group had a 2.92-percentage-point lower mean hop LSI than the no-injury group (*β* = −2.92, 95% CI: −3.74 to −2.11, *p* < 0.001). After further adjustment for sex, age, BMI, weekly training duration, training experience, activity group, and low back pain in the past 3 months, the association remained, with no attenuation in the effect estimate (*β* = −3.13, 95% CI: −3.86 to −2.40, *p* < 0.001). In the final model, after additional adjustment for ankle dorsiflexion asymmetry, YBT mean reach asymmetry, and side-bridge asymmetry, past 12-month sports injury history remained associated with lower hop LSI. The injury group had a 2.48-percentage-point lower hop LSI than the no-injury group (*β* = −2.48, 95% CI: −3.29 to −1.66, *p* < 0.001). This finding indicates that the association between injury history and lower hop LSI remained stable after simultaneous consideration of demographic characteristics, training exposure, low back pain status, and lower-limb functional asymmetry measures (Table 3).

**Table 3:** Multivariable linear regression models for hop limb symmetry index.

Characteristic	Model 1: *β* (95% CI)	Model 1: *p* value	Model 2: *β* (95% CI)	Model 2: *p* value	Model 3: *β* (95% CI)	Model 3: *p* value
Injury history: injury vs. no injury	−2.92 (−3.74 to −2.11)	<0.001	−3.13 (−3.86 to −2.40)	<0.001	−2.48 (−3.29 to −1.66)	<0.001
Sex: male vs. female			−0.21 (−0.85 to 0.44)	0.481	−0.08 (−0.69 to 0.54)	0.781
Age, years			0.07 (−0.19 to 0.33)	0.560	0.07 (−0.18 to 0.31)	0.558
BMI, kg/m^2^			−0.04 (−0.21 to 0.13)	0.586	−0.06 (−0.24 to 0.12)	0.460
Training hours/week			0.08 (−0.08 to 0.25)	0.285	0.09 (−0.12 to 0.29)	0.363
Training years			0.01 (−0.25 to 0.26)	0.949	0.03 (−0.21 to 0.28)	0.774
Activity group: sports major vs. regular active			0.33 (−0.70 to 1.35)	0.482	0.09 (−1.27 to 1.45)	0.881
Low back pain in past 3 months: yes vs. no			−0.60 (−1.62 to 0.42)	0.211	−0.50 (−1.58 to 0.57)	0.314
Ankle asymmetry, %					−0.04 (−0.11 to 0.02)	0.148
YBT asymmetry, mean, cm					−0.11 (−0.43 to 0.22)	0.462
Side bridge asymmetry, %					−0.05 (−0.12 to 0.03)	0.177
Model fit statistics						
*n*	263		263		263	
*R* ^2^	0.203		0.228		0.262	
Adjusted *R*^2^	0.200		0.204		0.230	
AIC	1222.3		1227.8		1222.0	

Ordinary least squares linear regression was used. The intercept is not displayed. Values are *β* coefficients with 95% confidence intervals and *p* values. Complete-case analysis was performed separately for each model. Reference groups: injury history = No injury; sex = Female; activity group = Regular Active; low back pain in past 3 months = No. Standard error method: Model 1: Cluster-robust standard errors by class_id; clusters = 9; Model 2: Cluster-robust standard errors by class_id; clusters = 9; Model 3: Cluster-robust standard errors by class_id; clusters = 9. Blank cells indicate variables not included in the corresponding model.

**Figure 3:**
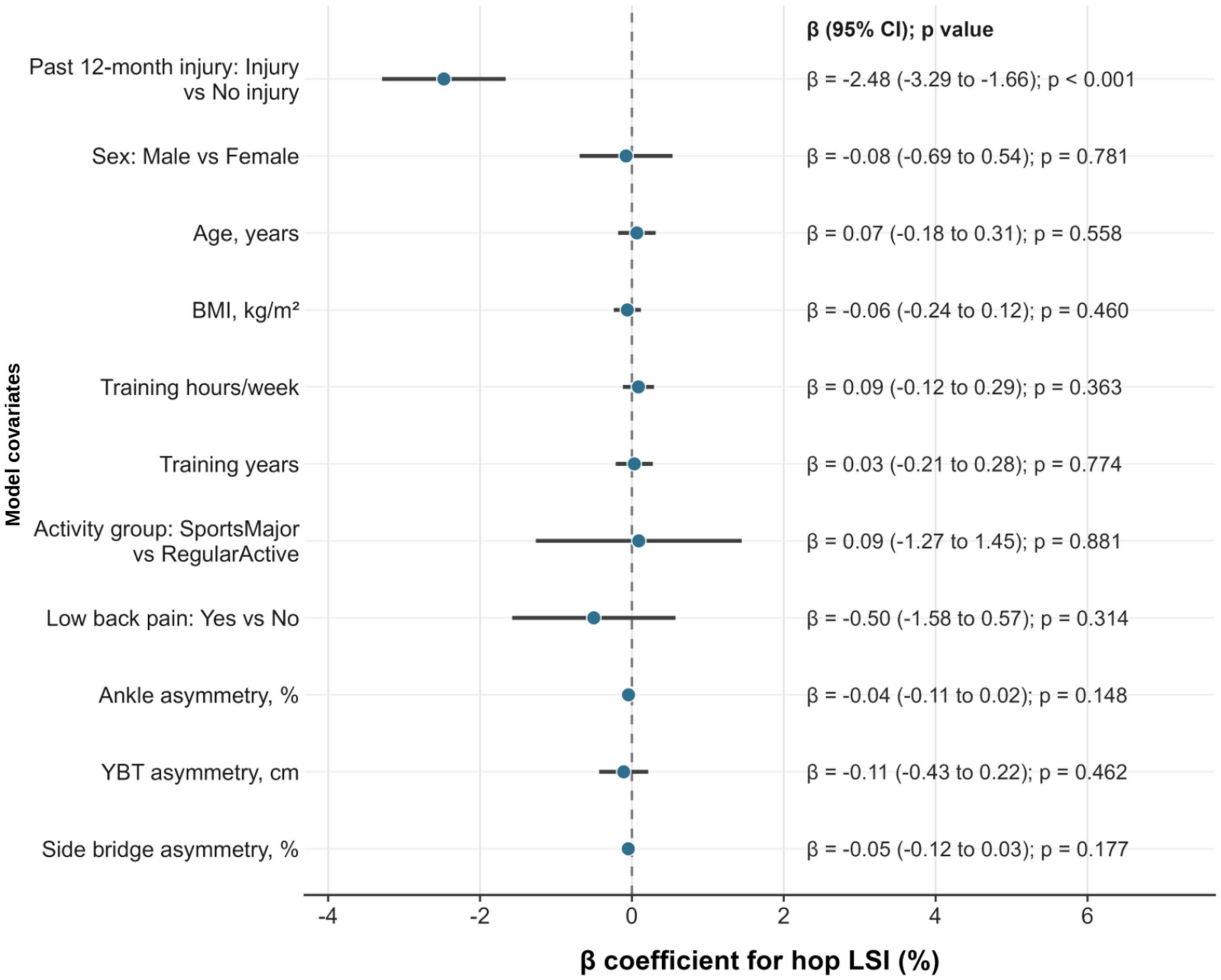
Adjusted associations with hop limb symmetry index.

Figure 3 presents the regression coefficients from the final multivariable model as a forest plot. The full model included 263 participants and used cluster-robust standard errors based on class_id, with 9 clusters. In the forest plot, past 12-month sports injury history was the most clearly directed variable in the final model, with a confidence interval that did not cross zero. In contrast, ankle dorsiflexion asymmetry, YBT mean reach asymmetry, and side-bridge asymmetry did not show independent statistical associations with hop LSI after simultaneous adjustment.

Multicollinearity diagnostics did not indicate serious collinearity in the fully adjusted model (Appendix 1). All VIF values were below 5, ranging from 1.027 to 3.782. The highest value was observed for activity group, but it remained below the prespecified threshold. Injury history also showed a low VIF value of 1.547. Spearman correlations among ankle dorsiflexion asymmetry, YBT mean reach asymmetry, side-bridge asymmetry, and hop LSI were weak, with absolute rho values ranging from 0.054 to 0.214. These findings suggest that the included asymmetry variables did not show substantial redundancy.

### 3.4 Secondary binary outcomes and sensitivity analyses

Sensitivity analyses showed that the association between past 12-month sports injury history and lower hop LSI was consistent across different model specifications. In the primary full model, injury history was associated with lower hop LSI (*β* = −2.48, 95% CI: −3.29 to −1.66, *p* < 0.001). After exclusion of outlier-flagged observations, the analysis included 250 participants, and the effect estimate remained close to that of the primary model (*β* = −2.54, 95% CI: −3.28 to −1.80, *p* < 0.001). After additional adjustment for core_composite_z, the association also remained stable (*β* = −2.52, 95% CI: −3.42 to −1.63, *p* < 0.001). These findings suggest that the association between injury history and lower hop LSI was not materially affected by exclusion of outliers or additional adjustment for the core composite function measure (Table 4).

**Table 4:** Secondary and sensitivity analyses.

Analysis	Outcome	Model type	*n*	Events	Effect estimate	95% CI	*p* value	Interpretation
A1. Primary full model	hop_lsi_mean_pct	OLS linear regression; cluster-robust SE by class_id; clusters = 9	263	Not applicable	*β* = −2.48	−3.29 to −1.66	<0.001	Injury was associated with lower hop LSI compared with no injury.
A2. Outlier-excluded model	hop_lsi_mean_pct	OLS linear regression; cluster-robust SE by class_id; clusters = 9	250	Not applicable	*β* = −2.54	−3.28 to −1.80	<0.001	Injury was associated with lower hop LSI compared with no injury.
A3. Core-adjusted sensitivity model	hop_lsi_mean_pct	OLS linear regression; cluster-robust SE by class_id; clusters = 9	263	Not applicable	*β* = −2.52	−3.42 to −1.63	<0.001	Injury was associated with lower hop LSI compared with no injury.
B1. Low LSI < 92%	hop_lsi_lt92	Logistic regression; cluster-robust SE by class_id; clusters = 9	263	42	OR = 13.55	3.19 to 57.47	0.003	Injury was associated with higher odds of the low-LSI outcome compared with no injury.
B2. Low LSI < 90%	hop_lsi_lt90	Logistic regression; cluster-robust SE by class_id; clusters = 9	263	18	OR = 11.04	3.87 to 31.55	<0.001	Injury was associated with higher odds of the low-LSI outcome compared with no injury.
C1. injury_12m × sex	hop_lsi_mean_pct	OLS linear interaction test; cluster-robust SE by class_id; clusters = 9	263	Not applicable	Interaction *p* value only		0.245	No clear evidence that the injury-LSI association differs by sex.
C2. injury_12m × activity_group	hop_lsi_mean_pct	OLS linear interaction test; cluster-robust SE by class_id; clusters = 9	263	Not applicable	Interaction *p* value only		0.787	No clear evidence that the injury-LSI association differs by activity group.

Complete-case analysis was performed separately for each analysis. Linear models used ordinary least squares regression. Effect estimates for linear models are *β* coefficients for injury compared with no injury. Logistic models report odds ratios for injury compared with no injury. For linear models, standard errors were cluster-robust by class_id when available with more than one cluster; otherwise HC3 robust standard errors were used. For regular logistic regression, robust or cluster-robust standard errors were used when applicable. If separation or non-convergence was detected, Firth logistic regression was used with model-based profile penalized-likelihood confidence intervals. Events indicate the number of participants meeting the binary low-LSI outcome; events are not applicable for linear models and interaction tests.

When low LSI was analysed as a binary outcome, past 12-month sports injury history was also associated with higher odds of low LSI. For the LSI < 92% outcome, 42 events were observed among 263 participants. In the main covariate-adjusted standard logistic model, injury history was associated with higher odds of LSI < 92% (OR = 17.52, 95% CI: 7.07–48.45, *p* < 0.001). After additional exploratory adjustment for ankle dorsiflexion asymmetry, YBT mean reach asymmetry, and side-bridge asymmetry, the association remained consistent (OR = 13.55, 95% CI: 3.19 to 57.47, *p* = 0.003). For the more stringent LSI < 90% threshold, 18 events were observed among 263 participants. Injury history was associated with higher odds of LSI < 90% in the main covariate-adjusted standard logistic model (OR = 21.86, 95% CI: 6.13–97.64, *p* < 0.001) and in the exploratory functional-adjusted model (OR = 11.04, 95% CI: 3.87–31.55, *p* < 0.001). Because of the limited number of events, especially for LSI < 90%, these threshold-based analyses were interpreted as secondary sensitivity evidence rather than as primary evidence. Supplementary Figure S2 displays the sensitivity analyses for the linear and logistic models as a forest plot and reports the sample size for each model and the number of events for the binary outcomes.

Interaction analyses did not show clear evidence of effect modification. The *p* value for the injury_12m × sex interaction was 0.245, and the p value for the injury_12m × activity_group interaction was 0.787. Overall, the sensitivity analyses and binary outcome analyses were consistent with the direction of the primary analysis, and no clear evidence was observed that the association between injury history and hop LSI differed by sex or activity group.

Study-size and small-event sensitivity analyses were conducted to contextualize the robustness of the primary continuous and secondary binary findings. For the primary continuous outcome, the available sample included 202 participants without past 12-month injury history and 61 participants with past 12-month injury history. The observed mean difference in hop LSI was −2.92 percentage points (95% CI: −3.89 to −1.96, *p* < 0.001), which was larger than the *post hoc* minimum detectable absolute difference of 1.01 percentage points. The corresponding Cohen’s *d* was −1.19, whereas the minimum detectable Cohen’s *d* was 0.41.

For the secondary binary low-LSI outcomes, 42 events were observed for LSI < 92% and 18 events for LSI < 90%, indicating limited event numbers, especially for the more stringent LSI < 90% threshold. Therefore, Firth penalized logistic regression was used as a small-event sensitivity analysis. In the main covariate-adjusted Firth models, past 12-month sports injury history remained associated with higher odds of LSI < 92% (Firth OR = 14.79, 95% CI: 6.20–39.02, *p* < 0.001) and LSI < 90% (Firth OR = 16.34, 95% CI: 5.01–64.50, *p* < 0.001). After additional exploratory adjustment for ankle dorsiflexion asymmetry, YBT mean reach asymmetry, and side-bridge asymmetry, the corresponding Firth estimates remained consistent for both LSI < 92% (Firth OR = 11.17, 95% CI: 4.18–32.79, *p* < 0.001) and LSI < 90% (Firth OR = 8.28, 95% CI: 2.22–36.62, *p* = 0.001). These findings were consistent in direction with the standard logistic models and supported the small-event robustness of the secondary threshold-based analyses. However, because these binary outcomes were based on thresholds and included limited event numbers, they were interpreted as secondary sensitivity evidence rather than as primary evidence.

### 3.5 Correlation matrix and model diagnostics

Supplementary correlation analyses showed that bivariate correlations between hop LSI and individual lower-limb asymmetry measures were generally weak. In the Spearman correlation matrix, hop LSI was weakly negatively correlated with ankle dorsiflexion asymmetry (rho = −0.21). The correlations of hop LSI with YBT mean reach asymmetry and side-bridge asymmetry were small and were not clearly evident after Holm correction. In contrast, lower-extremity function *z*-score showed more concentrated correlations with hop performance measures, including single hop, triple hop, and crossover hop, indicating internal consistency among functional performance measures (Supplementary Table S2).

Diagnostic plots for the final ordinary least squares (OLS) model are presented in Supplementary Figure S1. The full model included 263 participants and estimated 12 predictors. The residuals-versus-fitted plot showed that residuals were generally distributed around zero. The Q–Q plot showed acceptable fit in the central portion, with mild tail deviation. The scale-location plot did not suggest marked systematic heteroscedasticity. The Cook’s distance/leverage plot identified a small number of leverage points, but there was no indication that a single observation dominated the primary findings. Overall, the supplementary correlation analyses and model diagnostics were consistent with the primary analytical findings.

## 4 Discussion

This study examined the association between past 12-month sports injury history and hop limb symmetry in physically active university students and characterized the accompanying lower-limb and trunk functional asymmetry profile. Students with a past 12-month injury history showed lower hop LSI, a higher proportion of low LSI, and greater ankle dorsiflexion asymmetry, YBT mean reach asymmetry, and side-bridge asymmetry. The association between injury history and lower hop LSI remained stable after adjustment for demographic characteristics, training exposure, activity background, low back pain, and functional asymmetry measures, and was further supported by sensitivity analyses excluding outliers, additionally adjusting for the core composite measure, and using binary low-LSI outcomes. Given the cross-sectional and descriptive nature of the study, these findings should not be interpreted as evidence that past injury directly caused lower hop LSI or that the current screening battery can predict future injury at the individual level. Rather, they suggest that participants with a broadly defined past 12-month injury history showed a multidimensional current functional asymmetry profile that may be relevant for post-injury monitoring in university physical education and training settings. This finding should not be interpreted as evidence specific to any injury location, injury type or diagnosis, severity level, recovery stage, or rehabilitation status.

This finding is consistent with previous work on residual functional deficits after sports injury. Current return-to-sport and post-injury rehabilitation frameworks no longer rely solely on time since injury or symptom resolution, but instead emphasize multidimensional indicators such as strength, hop performance, balance, movement quality, and psychological readiness, because resuming training does not necessarily indicate full functional recovery (13, 21). In studies of anterior cruciate ligament reconstruction and lower-limb injury, return to sport without meeting objective functional criteria has been associated with higher reinjury risk, whereas delaying return and meeting functional testing requirements may reduce subsequent residual functional asymmetry (22, 23). Although the present study did not involve a postoperative rehabilitation population and was not restricted to a single injury type, the findings reflect a similar issue: even after individuals have returned to regular physical activity, past injury may still be associated with detectable bilateral functional differences. Hop LSI is particularly relevant in this context because it is not merely a static strength measure, but a field-based functional performance indicator integrating single-leg support, propulsion, landing attenuation, and movement confidence. Previous systematic reviews also suggest that single-leg hop tests can provide information on knee-related outcomes and recovery status after lower-limb injury (24, 25). Accordingly, the lower hop LSI observed in the injury group should not be viewed as an isolated statistical difference, but as a current bilateral performance difference observed in a heterogeneous injury-history group. Because injury location, injury type or diagnosis, severity, time since injury, and rehabilitation status were not available for stratified analysis, this finding cannot be interpreted as evidence of incomplete recovery from a specific injury.

A possible explanation is that some past injuries may be accompanied by residual deficits in force production, sensorimotor control, joint mobility, dynamic balance, trunk endurance, or movement confidence. However, because the injury-history variable combined heterogeneous injuries, these mechanisms remain hypothetical and cannot be assigned to specific anatomical locations, injury diagnoses, severities, recovery stages, or rehabilitation exposures in the present study. These deficits may not be fully apparent after students return to regular training, but they can emerge during integrated single-leg tasks such as hopping. Therefore, lower hop LSI and accompanying asymmetry measures may reflect a residual functional vulnerability rather than isolated test differences. However, because this study was cross-sectional, these mechanisms remain interpretive and require confirmation in longitudinal research.

The present study also found more evident differences in ankle dorsiflexion asymmetry and YBT mean reach asymmetry in the injury group, which is consistent with research on dynamic postural control and distal joint mobility. YBT reach asymmetry has been used to identify potential residual functional asymmetry in athletes, and limited ankle dorsiflexion range of motion has been shown to influence dynamic balance performance (15, 26). Models of chronic ankle instability further suggest that residual problems after an initial injury are often not attributable to a single structural deficit, but rather reflect the combined influence of joint mobility, sensorimotor control, perceived stability, and movement behavior (27). From this perspective, the greater ankle dorsiflexion asymmetry and YBT asymmetry observed in the injury group may reflect residual limitations in distal mobility and dynamic postural control. The effect size for side-bridge asymmetry was smaller, but the finding remains informative. The side-bridge test is not equivalent to a hop task, yet it provides complementary information on trunk lateral endurance and side-to-side control differences. The present study did not show that any single asymmetry measure independently explained lower hop LSI; rather, these measures collectively described a broader functional asymmetry profile accompanying past injury.

Although LSI, ankle dorsiflexion asymmetry, YBT reach asymmetry, and side-bridge asymmetry were entered as separate variables in the regression model, they may be better understood as complementary components of an integrated functional asymmetry profile. Hop LSI reflects dynamic single-leg performance, ankle dorsiflexion asymmetry reflects distal mobility, YBT asymmetry reflects dynamic postural control, and side-bridge asymmetry reflects trunk lateral endurance. Recent work on chronic ankle instability has proposed feature-fusion and machine-learning frameworks to integrate neuromuscular and biomechanical features under different landing conditions, suggesting that multidimensional data may improve the identification of injury-related functional deficits (46). However, the present study was not designed to develop such a predictive model. Instead, it provides preliminary field-based evidence supporting a profile-oriented interpretation of functional asymmetry in physically active university students.

This study also extends previous literature in several respects. Existing evidence on hop LSI and return-to-sport assessment has largely come from anterior cruciate ligament injury or reconstruction populations and is frequently based on high-level athletes or single-sport teams. In contrast, the present study focused on physically active university students, including sports-major students and students with long-term exercise habits. This population lies between general young adults and competitive athletes, with substantial sport exposure but not necessarily systematic post-injury rehabilitation assessment. In this group, the more common practical question is not whether an individual can return to professional competition, but whether unrecognized functional asymmetry persists after resumption of daily training, coursework, and sport participation. The present study used past 12-month sports injury history as the exposure, rather than current pain, postoperative status, or a single test result, and used hop LSI as the primary outcome while also characterizing ankle dorsiflexion, YBT, and side-bridge asymmetry. This design more closely reflects real-world screening conditions in physical education and university sport training. Notably, in the final model, ankle dorsiflexion asymmetry, YBT mean reach asymmetry, and side-bridge asymmetry did not show independent associations with hop LSI after simultaneous adjustment, whereas injury history remained stably associated with lower hop LSI. This should not be interpreted as evidence that these asymmetry measures are unimportant. A more appropriate interpretation is that hop LSI may capture an integrated residual functional profile after past injury, including strength, coordination, landing control, joint mobility, postural strategy, and movement behavior, rather than being fully explained by any single asymmetry measure.

From the perspective of training science and neuromuscular control, the association between past injury history and lower hop LSI is plausible. Single-leg hopping is a highly integrated task that requires the lower limb to complete propulsion, take-off, landing attenuation, postural re-stabilization, and continuous control within a short time frame. Even after pain has resolved or an individual has returned to sport participation, residual differences may persist in force production, eccentric control, joint mobility, sensorimotor feedback, landing strategy, and single-leg support stability. Studies after anterior cruciate ligament reconstruction have shown that landing biomechanics and postural stability are associated with subsequent residual functional asymmetry, suggesting that recovery should not be evaluated only by local strength or joint mobility but should also consider overall control quality during dynamic tasks (14). LSI itself also has limitations: when both limbs are deconditioned or the uninvolved limb has not fully returned to normal function, LSI may overestimate functional recovery (7). In the present study, however, the association between injury history and hop LSI remained stable across multiple adjustments and sensitivity analyses, indicating that hop LSI still provided meaningful information on bilateral function in this sample. Ankle dorsiflexion asymmetry and YBT asymmetry can reflect distal mobility and dynamic postural control, but they do not fully reproduce the demands of impulse generation, rapid center-of-mass control, and landing attenuation during hopping; side-bridge asymmetry primarily reflects trunk lateral endurance rather than the complete process of hop control. Perspectives from motor learning and neuroplasticity also suggest that recovery after injury requires task-specific sensorimotor-cognitive retraining rather than relying solely on improvement in isolated functional measures (28).

Psychological and training-behavioral factors may also contribute to this phenomenon. The injury group had not only a greater burden of past injury but also a more concentrated sports-major background and higher training exposure. These baseline differences provided the basis for subsequent multivariable adjustment. Sports injury may affect not only tissue structure or physical function but also confidence in the injured limb, fear of reinjury, movement avoidance, and effort allocation. Systematic reviews have shown that psychological factors are closely related to return to sport after injury, and insufficient psychological readiness may limit return to previous sport levels (29, 30). Fear of reinjury and kinesiophobia do not necessarily lead to complete sport avoidance; they may also modify movement strategies in subtler ways, such as reducing load on one limb, shortening stance time, lowering willingness to generate propulsion, or adopting protective landing patterns (31, 32). Among university students and sports-major students, these psychological factors may interact with coursework, training schedules, assessment demands, and team participation pressure. Students may return to exercise earlier because of course requirements, training arrangements, or athletic identity, but return to participation does not necessarily indicate completion of adequate functional retraining. Training-load research suggests that residual functional asymmetry is not simply a linear function of workload magnitude, but depends more on the match between load progression, recovery capacity, and training adaptation (33, 34). If students lack progressive retraining in single-leg hopping, landing control, dynamic balance, and trunk control after injury, subtle asymmetry may persist despite their ability to complete daily sport activities. In physical education practice, emphasis is often placed on whether students can complete a movement, course task, or performance standard, whereas bilateral symmetry and movement quality are less consistently monitored. Studies in physical education have shown that motor competence and the quality of classroom physical activity require intentional pedagogical design and do not emerge solely from participation (35, 36). Therefore, the functional asymmetry observed in this study should not be attributed to a single psychological or biomechanical factor; it is more likely to reflect the combined influence of injury experience, training recovery, task exposure, and the teaching environment.

These findings have applied implications for training, teaching, and post-injury functional monitoring in physically active university students, but they should be interpreted as descriptive rather than predictive. As a simple and feasible field-based assessment measure, hop LSI may be used to describe persistent bilateral hop performance differences among groups of students with past 12-month injury history. It should not be used alone as evidence for return to sport, functional recovery, or future injury risk. Instead, it can be considered alongside ankle dorsiflexion asymmetry, YBT reach asymmetry, and side-bridge asymmetry to provide a broader functional assessment profile. For sports-major students and students who regularly engage in exercise, asking whether pain has resolved or whether training has resumed may be insufficient to describe functional recovery. A more appropriate approach is to incorporate bilateral functional symmetry checks into coursework, training, and recovery processes, with attention to single-leg hopping, landing control, ankle mobility, dynamic balance, and trunk lateral control. For students reporting past 12-month sports injury history, continued functional monitoring may be considered rather than assuming complete recovery based only on return to participation (44, 45). In university physical education and training practice, post-injury functional assessment can be integrated into routine teaching and training processes to document students’ bilateral functional quality after return to participation.

Because of the cross-sectional design, this study cannot determine the temporal direction between injury history and functional asymmetry. The observed lower hop LSI and greater asymmetry may reflect residual deficits after previous injury, pre-existing functional characteristics that increased susceptibility to injury, or both. Therefore, these findings should be interpreted as evidence of an association between past injury history and current functional asymmetry, rather than as proof of causality or prospective injury risk. Longitudinal studies with baseline functional assessment and subsequent injury surveillance are needed to clarify the temporal and predictive roles of these measures.

A feasible next step would be to assess hop LSI, ankle dorsiflexion asymmetry, YBT reach asymmetry, and side-bridge asymmetry at baseline, follow students across a semester or training season, and record new injuries, recovery status, and changes in functional performance. Such a design would help determine whether these asymmetry indicators represent residual consequences of previous injury, pre-existing susceptibility factors, or modifiable markers of recovery.

This study has several limitations. First, the cross-sectional design does not allow inference about causality or temporal sequence between past 12-month sports injury history and lower hop LSI. Second, the primary exposure was a broad binary indicator of any past 12-month sports injury history, which may have combined injuries differing in anatomical location, injury type or diagnosis, severity, time since injury, and rehabilitation or return-to-sport status. Although injured-side information was summarized descriptively and exploratorily when available, it cannot replace detailed stratification by injury location, type, severity, recovery stage, or rehabilitation exposure. Therefore, the findings should be interpreted as average associations for a heterogeneous injury-history group rather than evidence specific to any injury subtype. Future longitudinal studies should collect standardized injury-surveillance data and perform adequately powered stratified analyses. Third, the sample consisted of physically active university students, and caution is needed when generalizing the findings to sedentary populations, clinical rehabilitation populations, or high-level professional athletes. The binary LSI thresholds should be interpreted cautiously. Although LSI ≥ 90% is commonly used as a conventional functional symmetry benchmark, it has not been specifically validated as an injury-risk cutoff in physically active university students. The LSI < 92% threshold was exploratory and intended to capture milder asymmetry. Therefore, threshold-based findings were used only to support the descriptive interpretation of the continuous hop LSI results, rather than to define clinical impairment or predict future injury. Future longitudinal studies may consider using feature-fusion approaches, composite asymmetry scores, or machine-learning models to integrate hop performance, ankle mobility, dynamic balance, trunk endurance, and neuromuscular variables, and to evaluate whether such integrated profiles have longitudinal relevance for subsequent injury occurrence or recovery trajectories.

## 5 Conclusion

In this cross-sectional study, past 12-month sports injury history was associated with lower hop LSI and greater multidimensional functional asymmetry among physically active university students. These findings should be interpreted as descriptive evidence of current functional asymmetry rather than causal or predictive evidence. Because the injury exposure was broad and binary, the results cannot be interpreted as specific to injury location, injury type or diagnosis, severity, time since injury, rehabilitation status, or recovery stage. Combining hop LSI with complementary measures of ankle mobility, dynamic balance, and trunk lateral endurance may help characterize post-injury functional profiles and guide further functional monitoring after return to participation. Longitudinal studies with standardized injury surveillance are needed to determine the temporal sequence and predictive validity of these field-based measures.

## Data Availability

The raw data supporting the conclusions of this article will be made available by the authors, without undue reservation.
