# Supplementary figures and images for: Association of past 12-month sports injury history with hop limb symmetry index in physically active university students: a cross-sectional study of field-based functional asymmetry profiles
Source: Front Public Health. 2026 Jul 3;14:1868536. doi: 10.3389/fpubh.2026.1868536 (PMC13375735; doi:10.3389/fpubh.2026.1868536)

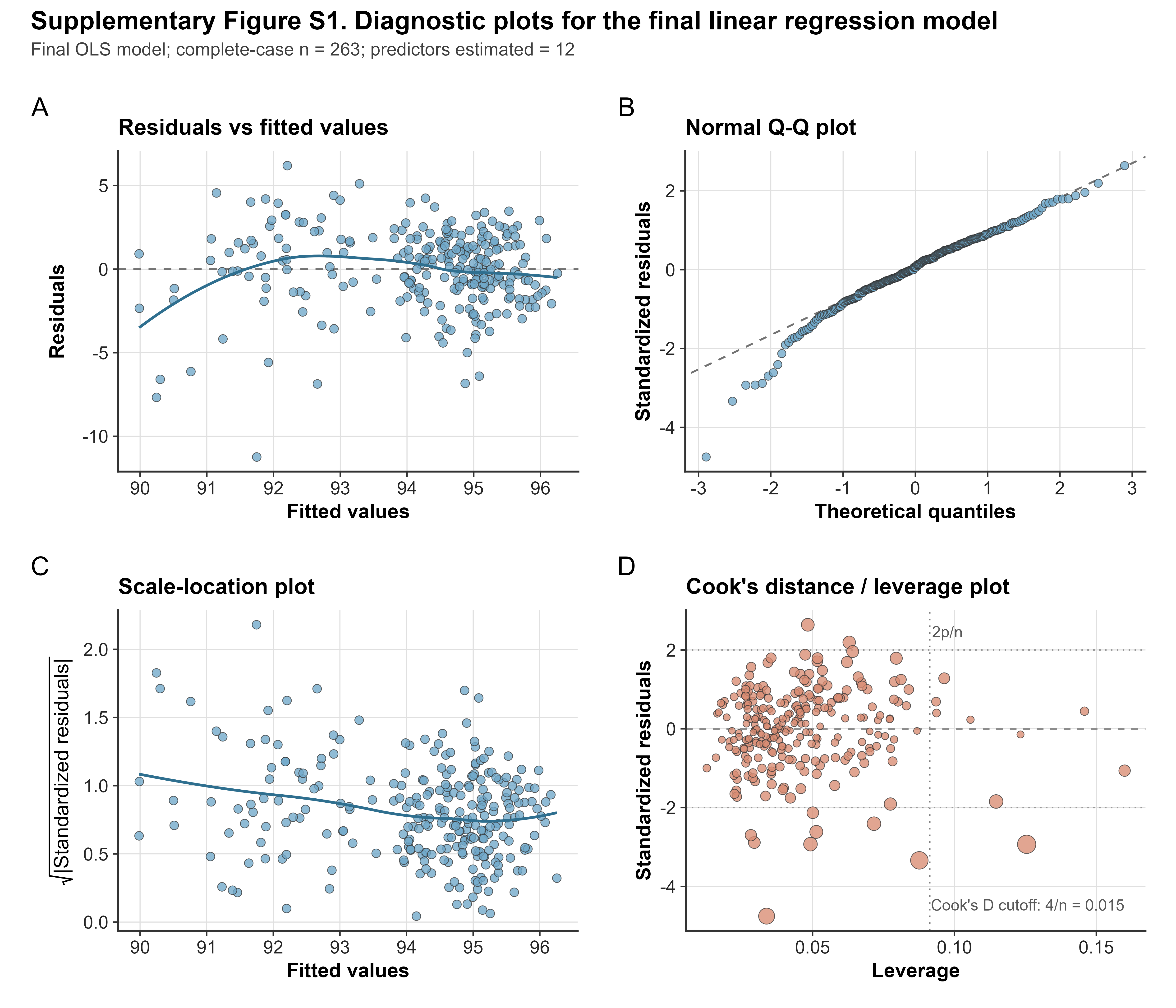

Supplement: Supplementary file 1 [file Image_1.TIFF]

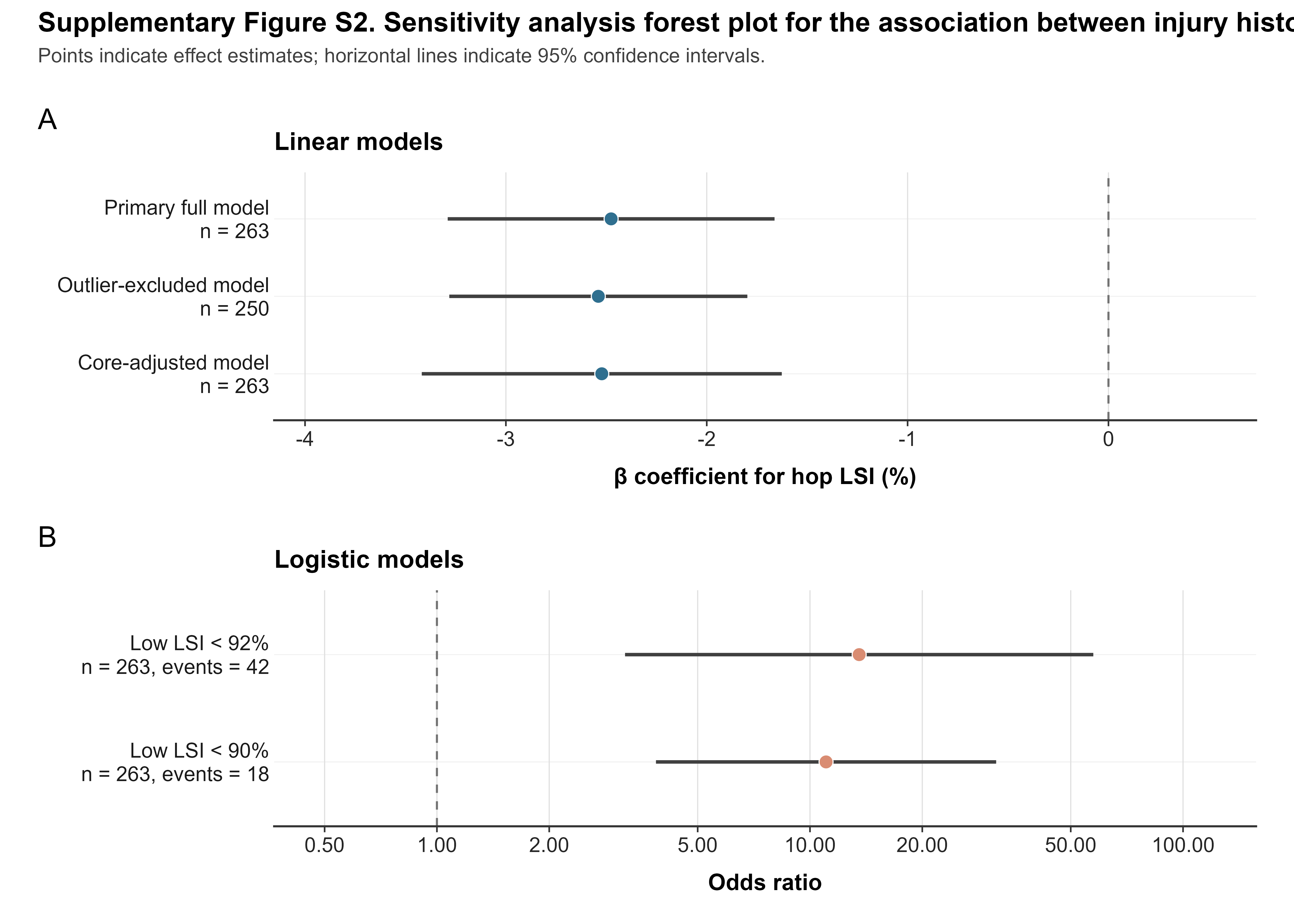

Supplement: Supplementary file 2 [file Image_2.TIFF]
