# Supplementary material for: Association of past 12-month sports injury history with hop limb symmetry index in physically active university students: a cross-sectional study of field-based functional asymmetry profiles
Source: Front Public Health. 2026 Jul 3;14:1868536. doi: 10.3389/fpubh.2026.1868536 (PMC13375735; doi:10.3389/fpubh.2026.1868536)
Supplement: Supplementary file 3 [file Table_1.docx]

# Supplementary Table S1. Variable definitions and coding scheme

| **Variable** | **Role in analysis** | **Definition** | **Coding / unit** | **Use in main analysis** |
| --- | --- | --- | --- | --- |
| participant_id | Identifier | Unique participant identifier. | Nominal identifier; not used as an analytical predictor. | Used for data tracking only; not included as a model covariate. Present in the source dataset. |
| sex | Participant characteristic / covariate | Participant sex recorded at assessment. | Categorical; coded according to source data and harmonized for analysis when needed. | Included as a descriptive variable and adjustment covariate. Present in the source dataset. |
| activity_group | Participant characteristic / covariate | Participant activity or training group. | Categorical; source-data group labels retained and used as factor levels. | Included as a descriptive variable and adjustment covariate. Present in the source dataset. |
| sports_major | Participant characteristic | Indicator of whether the participant was a sports major. | Binary or categorical indicator; source-data coding harmonized when needed. | Included in participant characteristics. Present in the source dataset. |
| program_track | Participant characteristic | Academic or training program track. | Categorical; source-data labels retained. | Included in participant characteristics. Present in the source dataset. |
| class_id | Clustering variable | Class or cluster identifier used for cluster-robust standard errors when available. | Categorical cluster identifier. | Used to calculate cluster-robust standard errors when available. Present in the source dataset. |
| age_years | Participant characteristic / covariate | Participant age at assessment. | Years. | Included as a descriptive variable and adjustment covariate. Present in the source dataset. |
| bmi | Participant characteristic / covariate | Body mass index. | kg/m². | Included as a descriptive variable and adjustment covariate. Present in the source dataset. |
| training_hours_week | Training exposure / covariate | Average weekly training duration. | Hours per week. | Included as a descriptive variable and adjustment covariate. Present in the source dataset. |
| training_years | Training history / covariate | Total years of training experience. | Years. | Included as a descriptive variable and adjustment covariate. Present in the source dataset. |
| injury_12m | Primary exposure | History of sports-related injury in the previous 12 months. | Binary; harmonized as No injury and Injury, with No injury as the reference group. | Primary exposure in group comparisons and regression models. Present in the source dataset. |
| injured_side | Injury descriptor | Side of the body affected by injury, when applicable. | Categorical; side affected, such as left, right, bilateral, or other source-data labels. | Used to describe injury characteristics when applicable. Present in the source dataset. |
| low_back_pain_3m | Clinical covariate | History of low back pain in the previous 3 months. | Binary; harmonized as No and Yes when needed. | Included as a descriptive variable and adjustment covariate. Present in the source dataset. |
| sim_outlier_flag | Sensitivity-analysis flag | Indicator of whether the participant was flagged as a simulation or data outlier. | Binary; 0/No = not flagged, 1/Yes = outlier flagged. | Used for outlier-excluded sensitivity analysis. Present in the source dataset. |
| ankle_asym_pct | Functional asymmetry measure | Relative ankle dorsiflexion asymmetry between limbs. | Percentage points (%). | Included in functional asymmetry profiles and adjusted models. Present in the source dataset. |
| ankle_asym_cm | Functional asymmetry measure | Absolute ankle dorsiflexion asymmetry between limbs. | Centimeters (cm). | Included in functional asymmetry profiles. Present in the source dataset. |
| ybt_asym_mean_cm | Functional asymmetry measure | Mean asymmetry in Y-Balance Test reach distance. | Centimeters (cm). | Included in functional asymmetry profiles and adjusted models. Present in the source dataset. |
| ybt_ant_asym_cm | Functional asymmetry measure | Anterior reach asymmetry in the Y-Balance Test. | Centimeters (cm). | Included in domain-specific YBT asymmetry summaries. Present in the source dataset. |
| ybt_pm_asym_cm | Functional asymmetry measure | Posteromedial reach asymmetry in the Y-Balance Test. | Centimeters (cm). | Included in domain-specific YBT asymmetry summaries. Present in the source dataset. |
| ybt_pl_asym_cm | Functional asymmetry measure | Posterolateral reach asymmetry in the Y-Balance Test. | Centimeters (cm). | Included in domain-specific YBT asymmetry summaries. Present in the source dataset. |
| side_bridge_asym_pct | Functional asymmetry measure | Relative asymmetry in side bridge performance between sides. | Percentage points (%). | Included in functional asymmetry profiles and adjusted models. Present in the source dataset. |
| hop_lsi_mean_pct | Primary continuous outcome | Mean hop limb symmetry index across hop tests. | Percentage (%). | Primary continuous outcome in linear regression models. Present in the source dataset. |
| hop_lsi_lt90 | Derived binary outcome | Indicator of hop limb symmetry index below 90%. | Derived binary variable; TRUE/1 if hop_lsi_mean_pct < 90, otherwise FALSE/0. | Secondary binary outcome for low hop LSI risk. Derived variable not present in the source dataset; defined as hop_lsi_mean_pct < 90. |
| hop_lsi_lt92 | Derived binary outcome | Indicator of hop limb symmetry index below 92%. | Derived binary variable; TRUE/1 if hop_lsi_mean_pct < 92, otherwise FALSE/0. | Secondary binary outcome for low hop LSI risk. Derived variable not present in the source dataset; defined as hop_lsi_mean_pct < 92. |
| hop_single_mean_cm | Functional performance measure | Mean single-hop distance. | Centimeters (cm). | Included in functional performance profiles. Present in the source dataset. |
| hop_triple_mean_cm | Functional performance measure | Mean triple-hop distance. | Centimeters (cm). | Included in functional performance profiles. Present in the source dataset. |
| hop_crossover_mean_cm | Functional performance measure | Mean crossover-hop distance. | Centimeters (cm). | Included in functional performance profiles. Present in the source dataset. |
| timed_6m_hop_mean_s | Functional performance measure | Mean time to complete the 6-m timed hop test. | Seconds (s). | Included in functional performance profiles. Present in the source dataset. |
| less_score | Movement-quality measure | Landing Error Scoring System score. | Score; higher values indicate more landing errors. | Included as a movement-quality measure. Present in the source dataset. |
| simple_reaction_time_ms | Neurocognitive performance measure | Simple reaction time. | Milliseconds (ms). | Included as a neurocognitive performance measure. Present in the source dataset. |
| lower_ext_function_z | Composite functional score | Standardized lower-extremity functional performance score. | Standardized z-score. | Included as a standardized functional performance index. Present in the source dataset. |
| global_function_z | Composite functional score | Standardized global functional performance score. | Standardized z-score. | Included as a standardized global performance index. Present in the source dataset. |
| core_composite_z | Core performance composite | Standardized composite score representing core performance. | Standardized z-score. | Included in core-adjusted sensitivity analysis. Present in the source dataset. |
| This supplementary table defines variables used in the main and sensitivity analyses. | | | | |
| The source dataset was read only to check whether listed variables were present. | | | | |
| If hop_lsi_lt90 or hop_lsi_lt92 was not present in the source dataset, it was described as a derived variable based on hop_lsi_mean_pct. | | | | |
