# Supplementary material for: Association of past 12-month sports injury history with hop limb symmetry index in physically active university students: a cross-sectional study of field-based functional asymmetry profiles
Source: Front Public Health. 2026 Jul 3;14:1868536. doi: 10.3389/fpubh.2026.1868536 (PMC13375735; doi:10.3389/fpubh.2026.1868536)
Supplement: Supplementary file 4 [file Table_2.docx]

# Supplementary Table S2. Correlation matrix of hop LSI, asymmetry indicators, and functional performance variables

| **Variable** | **Hop LSI** | **Ankle asym, %** | **Ankle asym, cm** | **YBT asym** | **Side bridge asym** | **LE function z** | **Global function z** | **YBT composite** | **Single hop** | **Triple hop** | **Crossover hop** | **Timed 6-m hop** | **LESS** | **Reaction time** |
| --- | --- | --- | --- | --- | --- | --- | --- | --- | --- | --- | --- | --- | --- | --- |
| Hop LSI (%) | — |  |  |  |  |  |  |  |  |  |  |  |  |  |
| Ankle asymmetry (%) | -0.21 (0.029) | — |  |  |  |  |  |  |  |  |  |  |  |  |
| Ankle asymmetry (cm) | -0.21 (0.040) | 1.00 (<0.001) | — |  |  |  |  |  |  |  |  |  |  |  |
| YBT mean asymmetry (cm) | -0.14 (1.000) | 0.12 (1.000) | 0.12 (1.000) | — |  |  |  |  |  |  |  |  |  |  |
| Side bridge asymmetry (%) | -0.14 (1.000) | -0.05 (1.000) | -0.06 (1.000) | 0.09 (1.000) | — |  |  |  |  |  |  |  |  |  |
| Lower-extremity function z-score | 0.14 (1.000) | -0.11 (1.000) | -0.10 (1.000) | -0.06 (1.000) | -0.13 (1.000) | — |  |  |  |  |  |  |  |  |
| Global function z-score | 0.13 (1.000) | -0.11 (1.000) | -0.10 (1.000) | -0.05 (1.000) | -0.06 (1.000) | 0.71 (<0.001) | — |  |  |  |  |  |  |  |
| YBT composite score (%) | 0.13 (1.000) | -0.14 (1.000) | -0.11 (1.000) | -0.01 (1.000) | -0.09 (1.000) | 0.70 (<0.001) | 0.60 (<0.001) | — |  |  |  |  |  |  |
| Single hop (cm) | 0.11 (1.000) | -0.07 (1.000) | -0.07 (1.000) | -0.03 (1.000) | -0.10 (1.000) | 0.88 (<0.001) | 0.58 (<0.001) | 0.47 (<0.001) | — |  |  |  |  |  |
| Triple hop (cm) | 0.11 (1.000) | -0.08 (1.000) | -0.09 (1.000) | -0.06 (1.000) | -0.14 (1.000) | 0.91 (<0.001) | 0.63 (<0.001) | 0.54 (<0.001) | 0.88 (<0.001) | — |  |  |  |  |
| Crossover hop (cm) | 0.12 (1.000) | -0.08 (1.000) | -0.08 (1.000) | -0.03 (1.000) | -0.15 (0.999) | 0.92 (<0.001) | 0.64 (<0.001) | 0.57 (<0.001) | 0.87 (<0.001) | 0.91 (<0.001) | — |  |  |  |
| Timed 6-m hop (s) | -0.13 (1.000) | 0.07 (1.000) | 0.07 (1.000) | 0.07 (1.000) | 0.10 (1.000) | -0.82 (<0.001) | -0.62 (<0.001) | -0.47 (<0.001) | -0.70 (<0.001) | -0.76 (<0.001) | -0.76 (<0.001) | — |  |  |
| LESS score | 0.02 (1.000) | 0.00 (1.000) | -0.01 (1.000) | 0.05 (1.000) | -0.00 (1.000) | -0.28 (<0.001) | -0.17 (0.393) | -0.17 (0.371) | -0.06 (1.000) | -0.03 (1.000) | -0.04 (1.000) | 0.06 (1.000) | — |  |
| Simple reaction time (ms) | -0.05 (1.000) | 0.06 (1.000) | 0.05 (1.000) | 0.02 (1.000) | -0.03 (1.000) | -0.26 (0.001) | -0.84 (<0.001) | -0.33 (<0.001) | -0.17 (0.338) | -0.21 (0.040) | -0.22 (0.022) | 0.24 (0.007) | 0.03 (1.000) | — |
| Values are Spearman correlation coefficients with Holm-adjusted p values in parentheses: rho (p value). | | | | | | | | | | | | | | |
| Only the lower triangle of the correlation matrix is shown. Diagonal cells are indicated by an em dash. | | | | | | | | | | | | | | |
| Spearman correlations were calculated using pairwise complete observations for each variable pair. | | | | | | | | | | | | | | |
| P values were adjusted for multiple testing using the Holm method across all pairwise correlations. | | | | | | | | | | | | | | |
