# Supplementary material for: Association of past 12-month sports injury history with hop limb symmetry index in physically active university students: a cross-sectional study of field-based functional asymmetry profiles
Source: Front Public Health. 2026 Jul 3;14:1868536. doi: 10.3389/fpubh.2026.1868536 (PMC13375735; doi:10.3389/fpubh.2026.1868536)
Supplement: Supplementary file 5 [file Table_3.docx]

## Supplementary Table S3. Exploratory regression analysis of hop LSI by injured side (reference: No injury)

| Model | Injury side group | β | 95% CI | P value | N |
| --- | --- | --- | --- | --- | --- |
| Model 1: Unadjusted | Left-side injury | -2.88 | -3.99, -1.77 | <0.001 | 263 |
| Model 1: Unadjusted | Right-side injury | -2.97 | -4.54, -1.39 | <0.001 | 263 |
| Model 2: Covariate-adjusted | Left-side injury | -3.12 | -4.23, -2.01 | <0.001 | 263 |
| Model 2: Covariate-adjusted | Right-side injury | -3.14 | -4.73, -1.54 | <0.001 | 263 |
| Model 3: Fully adjusted | Left-side injury | -2.54 | -3.77, -1.31 | <0.001 | 263 |
| Model 3: Fully adjusted | Right-side injury | -2.40 | -3.95, -0.86 | 0.002 | 263 |

Note: HC3 heteroscedasticity-robust standard errors were used (class_id had only 9 clusters, below the threshold of 10 for cluster-robust SE). Reference group: No injury. Model 1: unadjusted. Model 2: adjusted for sex, age, BMI, training hours/week, training years, activity group, and low back pain history. Model 3: additionally adjusted for ankle dorsiflexion asymmetry, YBT mean reach asymmetry, and side bridge asymmetry. This analysis is exploratory. The broad binary injury-history variable remains a limitation.
