# Supplementary material for: Association of past 12-month sports injury history with hop limb symmetry index in physically active university students: a cross-sectional study of field-based functional asymmetry profiles
Source: Front Public Health. 2026 Jul 3;14:1868536. doi: 10.3389/fpubh.2026.1868536 (PMC13375735; doi:10.3389/fpubh.2026.1868536)
Supplement: Supplementary file 6 [file Table_4.docx]

## Supplementary Table S4. Small-sample robustness analyses for the association between injury history and hop LSI

| **Model** | **Inference method** | **No. of clusters** | **Beta** | **SE** | **df** | **95% CI** | **P value** | **Interpretation** |
| --- | --- | --- | --- | --- | --- | --- | --- | --- |
| Model 2 | Conventional OLS SE | — | -3.13 | 0.38 | 254.0 | -3.88, -2.38 | <0.001 | Consistent with the main finding |
|  | HC3 robust SE | — | -3.13 | 0.50 | 254.0 | -4.11, -2.15 | <0.001 | Consistent with the main finding |
|  | Cluster-robust SE (class_id) | 9 | -3.13 | 0.32 | — | -3.76, -2.50 | <0.001 | Consistent with the main finding |
|  | CR2 + Satterthwaite correction | 9 | -3.13 | 0.32 | — | -3.88, -2.38 | <0.001 | Consistent with the main finding |
|  | Wild cluster bootstrap (class_id) | 9 | — | — | — | — | — | Not available |
| Model 3 | Conventional OLS SE | — | -2.48 | 0.44 | 251.0 | -3.34, -1.61 | <0.001 | Consistent with the main finding |
|  | HC3 robust SE | — | -2.48 | 0.53 | 251.0 | -3.52, -1.43 | <0.001 | Consistent with the main finding |
|  | Cluster-robust SE (class_id) | 9 | -2.48 | 0.35 | — | -3.17, -1.78 | <0.001 | Consistent with the main finding |
|  | CR2 + Satterthwaite correction | 9 | -2.48 | 0.35 | — | -3.30, -1.66 | <0.001 | Consistent with the main finding |
|  | Wild cluster bootstrap (class_id) | 9 | — | — | — | — | — | Not available |

Note: Model 2 was adjusted for sex, age, BMI, weekly training duration, training experience, activity group, and low back pain in the past 3 months. Model 3 additionally included ankle dorsiflexion asymmetry, YBT mean reach asymmetry, and side-bridge asymmetry and was interpreted as an exploratory functional-profile-adjusted model. Cluster-robust inference was based on class_id, with 9 clusters. Because the number of clusters was small, HC3 robust standard errors and CR2 cluster-robust standard errors with Satterthwaite small-sample correction were additionally used as robustness analyses. LSI, limb symmetry index; CI, confidence interval; SE, standard error.
