# Supplementary material for: Association of past 12-month sports injury history with hop limb symmetry index in physically active university students: a cross-sectional study of field-based functional asymmetry profiles
Source: Front Public Health. 2026 Jul 3;14:1868536. doi: 10.3389/fpubh.2026.1868536 (PMC13375735; doi:10.3389/fpubh.2026.1868536)
Supplement: Supplementary file 7 [file Table_5.docx]

## Supplementary Table S5. Study-size and small-event sensitivity analyses for hop LSI outcomes

### A. Post hoc precision/detectable-effect analysis for the primary continuous outcome

| **Outcome** | **No injury n** | **Injury n** | **No injury mean +/- SD** | **Injury mean +/- SD** | **Mean difference** | **95% CI** | **P value** | **Cohen's d** | **Min detectable Cohen's d** | **Min detectable absolute difference** |
| --- | --- | --- | --- | --- | --- | --- | --- | --- | --- | --- |
| hop_lsi_mean_pct | 202 | 61 | 94.88 +/- 1.97 | 91.96 +/- 3.63 | -2.92 | -3.89, -1.96 | <0.001 | -1.19 | 0.41 | 1.01 |

### B. Small-event logistic sensitivity analyses for low hop LSI outcomes

| **Outcome** | **Events / total** | **Model** | **Method** | **OR** | **95% CI** | **P value** | **Interpretation** |
| --- | --- | --- | --- | --- | --- | --- | --- |
| Hop LSI < 92% | 42 / 263 | L1: Main covariate-adjusted | Standard logistic | 17.52 | 7.07, 48.45 | <0.001 | Consistent with the main finding |
|  | 42 / 263 | L2: Firth, main covariate-adjusted | Firth penalized logistic | 14.79 | 6.20, 39.02 | <0.001 | Consistent with the main finding |
|  | 42 / 263 | L3: Exploratory functional-adjusted | Standard logistic | 13.55 | 4.82, 42.23 | <0.001 | Consistent with the main finding |
|  | 42 / 263 | L4: Firth, exploratory functional-adjusted | Firth penalized logistic | 11.17 | 4.18, 32.79 | <0.001 | Consistent with the main finding |
| Hop LSI < 90% | 18 / 263 | L1: Main covariate-adjusted | Standard logistic | 21.86 | 6.13, 97.64 | <0.001 | Consistent with the main finding |
|  | 18 / 263 | L2: Firth, main covariate-adjusted | Firth penalized logistic | 16.34 | 5.01, 64.50 | <0.001 | Consistent with the main finding |
|  | 18 / 263 | L3: Exploratory functional-adjusted | Standard logistic | 11.04 | 2.62, 56.26 | 0.002 | Consistent with the main finding |
|  | 18 / 263 | L4: Firth, exploratory functional-adjusted | Firth penalized logistic | 8.28 | 2.22, 36.62 | 0.001 | Consistent with the main finding |

Note: Because this study was based on an existing cross-sectional dataset, no formal a priori sample size calculation was performed before data collection. Part A presents a post hoc precision/detectable-effect analysis for the primary continuous outcome, hop LSI. Part B presents small-event logistic sensitivity analyses for binary low-LSI outcomes. Firth penalized logistic regression was used as a sensitivity analysis because the number of events was limited, especially for the LSI <90% outcome. Model L1/L2 adjusted for sex, age, BMI, weekly training duration, training experience, activity group, and low back pain in the past 3 months. Model L3/L4 additionally included ankle dorsiflexion asymmetry, YBT mean reach asymmetry, and side-bridge asymmetry and was interpreted as exploratory rather than causal.
